# Role of CD9 Sensing, AI, and Exosomes in Cellular Communication of Cancer

**DOI:** 10.23937/2469-570X/1410079

**Published:** 2023-05-20

**Authors:** Neda Baghban, Sai Priyanka Kodam, Mujib Ullah

**Affiliations:** 1 Institute for Immunity and Transplantation, Stem Cell Biology and Regenerative Medicine, School of Medicine, Stanford University, USA; 2 Molecular Medicine Department of Medicine, Stanford University, USA

**Keywords:** Exosome, Cell Communications and Signalling, Cancer, Metastasis, Exosomes, CD9, Artificial intelligence

## Abstract

Exosomes are small membrane-bound vesicles that are released by various types of cells, including cancer cells, and play a role in intercellular communication. CD9 is a protein that is involved in cell signaling and adhesion. It is found on the surface of various cells, including cancer cells, and has been implicated in the communication between cancer cells and their microenvironment. Exosomes are small membrane-bound vesicles that are released by cells and contain various bioactive molecules, such as proteins, lipids, and nucleic acids. Exosomes have been shown to play a role in intercellular communication, and they have been implicated in the progression of cancer. There is evidence to suggest that CD9 is involved in the packaging and release of exosomes by cancer cells. CD9 has been shown to be important for the formation of tetraspanin-enriched microdomains (TEMs) on the surface of exosomes. These TEMs are thought to be important for the sorting and packaging of specific molecules into exosomes. In summary, CD9 appears to play an important role in the communication between cancer cells and their microenvironment via exosomes. The precise mechanisms by which CD9 mediates this communication are still being investigated, but the involvement of CD9 in exosome packaging and uptake suggests that it may be a promising target for the development of novel cancer therapies. Furthermore, CD9 has been shown to be involved in the uptake of exosomes by recipient cells. For example, studies have shown that CD9-positive exosomes released by cancer cells can be taken up by other cancer cells, leading to the transfer of oncogenic molecules and the promotion of cancer progression.

## Introduction

Exosomes are small extracellular vesicles that are released by many types of cells, including cancer cells. They contain a variety of molecules, including proteins, lipids, and nucleic acids, and have been shown to play important roles in intercellular communication and the spread of cancer [1,2].

In cancer, exosomes can be involved in various processes that contribute to tumor progression, including promoting angiogenesis (the formation of new blood vessels to support the tumor), facilitating metastasis (the spread of cancer to other parts of the body), and suppressing the immune system [2–4]. Exosomes, a subset of EVs, have captured the attention of researchers for their ability to transport cell-specific cargo, including tumor-specific proteins and factors that establish pre-metastatic niches [5,6]. Exosomes have also been investigated as potential biomarkers for cancer diagnosis and prognosis [7,8]. Studies have shown that the content of exosomes released by cancer cells can differ from that of exosomes released by healthy cells, and that analyzing the molecular signature of these exosomes could potentially be used to detect cancer at an early stage [9–12]. Exosomes are tiny vesicles that are secreted by cells and contain various biomolecules such as proteins, RNA, and DNA [13,14]. They play an important role in cell-to-cell communication and have been studied for their potential use in diagnostic and therapeutic applications [14–16]. Another potential application of AI in the field of exosome research is in the development of exosome-based therapeutics [2,17,18]. Artificial intelligence (AI) can be used to design and optimize exosomes for specific therapeutic purposes, such as targeted drug delivery [2,19–21]. AI has been increasingly applied to the study of exosomes, particularly in the areas of exosome isolation, characterization, and analysis [2,22,23]. For example, AI algorithms can be used to analyze large datasets of exosome content and identify patterns and relationships that might be difficult for humans to detect [2,23]. Additionally, machine learning techniques can be used to predict the content of exosomes based on their physical properties, such as size and surface markers [2].

In the field of cancer research, AI has been used to analyze exosomes for biomarker discovery and diagnosis [24–26]. By analyzing the contents of exosomes isolated from cancer patients, researchers hope to identify specific molecules that could serve as early indicators of the disease or as targets for therapy [27,28]. The combination of exosome biology and AI holds great promise for advancing our understanding of intercellular communication and developing new diagnostic and therapeutic approaches for a wide range of diseases [2,29,30]. One potential application of AI in the field of exosome research is in the analysis of exosome data. As the field of exosome research grows, there is an increasing amount of data being generated, and AI can be used to analyze this data to identify patterns and relationships that may be difficult to detect using traditional methods [2,31,32].

Furthermore, researchers are exploring the potential of using exosomes as a tool for cancer treatment [33,34]. One approach involves engineering exosomes to carry therapeutic molecules, such as drugs or RNA-based therapies, to target cancer cells specifically [34,35]. Another approach involves using exosomes to deliver cancer vaccines or to stimulate the immune system to attack cancer cells [36]. However, much more research is needed to fully understand the role of exosomes in cancer and to develop effective exosome-based therapies.

## Cancer and Exosomes

Cancer cells interact with exosomes in various ways [37,38]. Cancer cells can release a higher number of exosomes than healthy cells, and the contents of these exosomes can promote tumour growth and progression [34,38]. Here are some ways in which cancer cells interact with exosomes, Exosomes secreted by cancer cells can promote tumour growth: Exosomes secreted by cancer cells can contain various molecules such as growth factors, cytokines, and oncogenes, which can be taken up by neighbouring cells or distant organs [34]. These molecules can promote the growth and proliferation of cancer cells and can also contribute to the formation of new blood vessels (angiogenesis) that feed the tumour [34,39].

**Figure 1:**
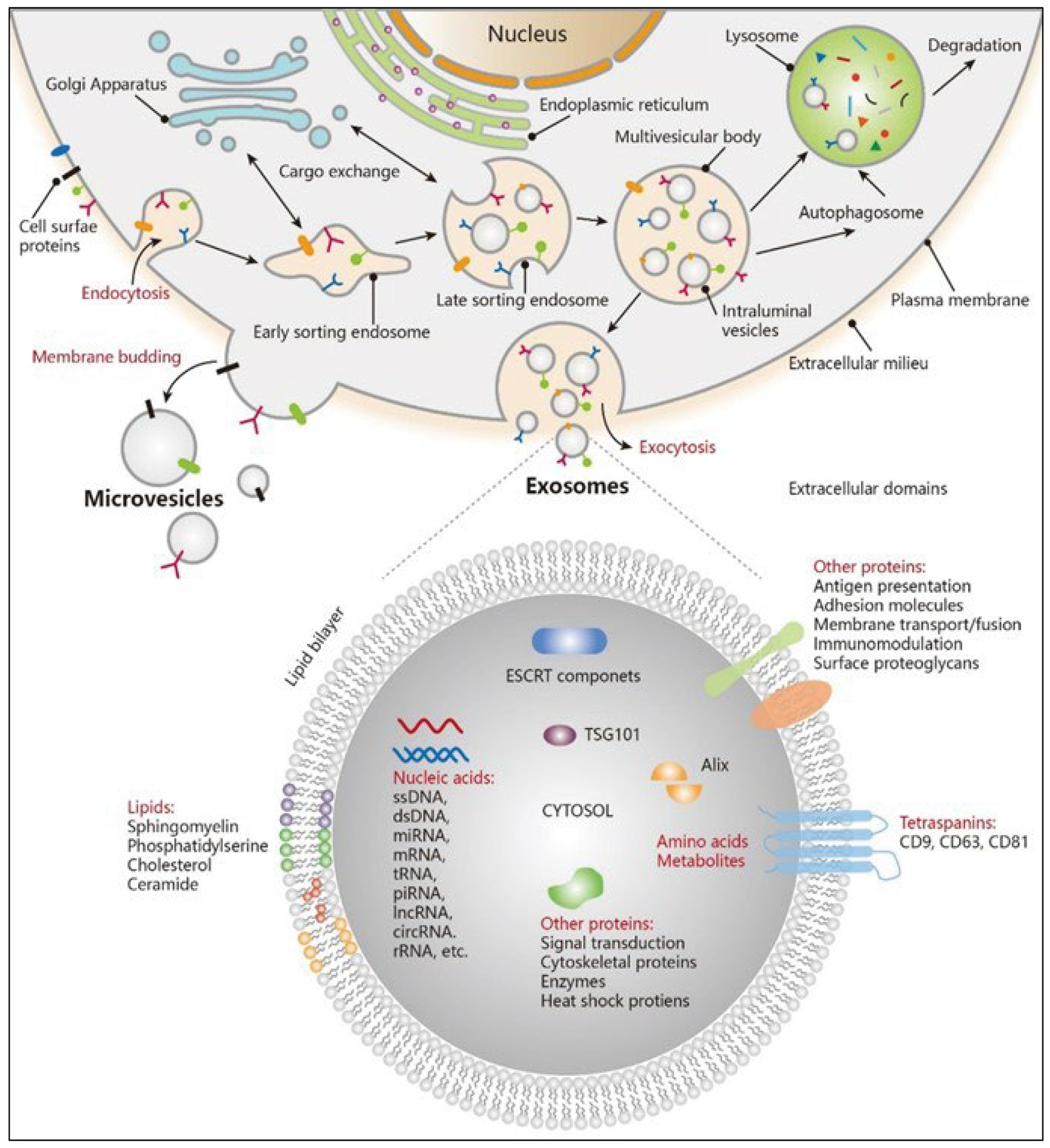
Exosome Biogenesis and its components (Adapted from 2021, American Chemical Society).

Exosomes can facilitate the spread of cancer cells: Cancer cells can release exosomes that contain molecules that can help to prepare the tumour microenvironment to be more receptive to metastasis [39]. For example, exosomes can carry enzymes that help to degrade the extracellular matrix surrounding the primary tumour, which can make it easier for cancer cells to invade and spread to other parts of the body [39] (Figure 1).

## Exosomes and Their Impact on Immune Suppression and Cancer Progression

Exosomes can suppress the immune system: Cancer cells can release exosomes that contain molecules that suppress the immune system, making it more difficult for the body to recognize and attack cancer cells [39,40]. These exosomes can contain proteins that inhibit the activity of immune cells such as T cells and can also carry microRNAs that regulate the expression of genes involved in immune function. Additionally, exosomes affect macrophages by promoting the production of M2 macrophages, which possess immunosuppressive and anti-inflammatory properties that facilitate tumour invasion and metastasis [40]. They also upregulate IL-10 and CD206 expression in naive macrophages, resulting in M2 polarization. Specific miRNAs carried by exosomes can further suppress macrophage immune function and contribute to cancer progression [39,41].

**Figure 2:**
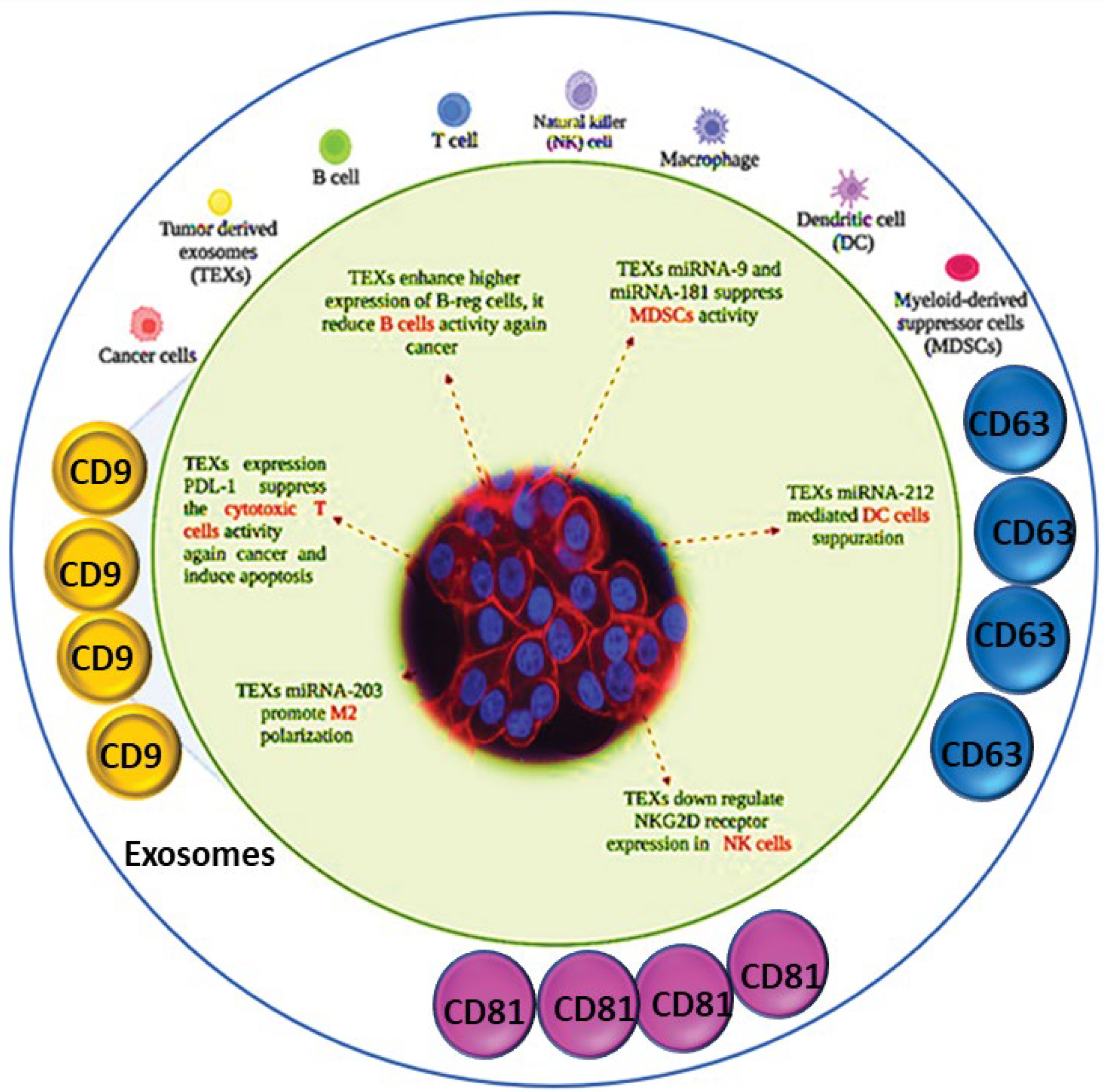
Role of CD9 in exosome communication with cancer and immune system.

Moreover, exosomes disrupt the maturation of immature myeloid cells (IMCs) into dendritic cells and monocytes, leading to an increase in myeloid-derived suppressor cells (MDSCs) with potent immunosuppressive activity [23,41,42]. Exosomes can inhibit the activity of immune cells: Cancer cells can release exosomes that contain molecules that inhibit the activity of immune cells such as T cells, B cells, and natural killer (NK) cells [4,5]. These exosomes can contain proteins such as programmed death-ligand 1 (PD-L1) that interact with receptors on immune cells and suppress their activity [4,5]. Exosomes can also carry microRNAs that regulate the expression of genes involved in immune function [3]. Exosomes can promote the activity of immunosuppressive cells: Cancer cells can release exosomes that contain molecules that promote the activity of immunosuppressive cells such as regulatory T cells (Tregs) and myeloid-derived suppressor cells (MDSCs) [5]. These cells can inhibit the activity of other immune cells and promote a tumour-supportive environment [5]. The interaction between cancer cells and exosomes is complex and can contribute to various aspects of tumour growth and progression [3,5]. Understanding the mechanisms underlying these interactions is important for developing new diagnostic and therapeutic strategies for cancer [3]. Exosomes can interfere with antigen presentation: Exosomes released by cancer cells can interfere with antigen presentation, which is the process by which immune cells recognize and respond to foreign substances such as tumour antigens [3]. For example, exosomes can carry molecules that downregulate the expression of major histocompatibility complex (MHC) molecules on cancer cells, which are necessary for antigen presentation [3,43]. Understanding the role of exosomes in regulating the immune response to cancer is important for developing new immunotherapies that can boost the immune response and improve outcomes for cancer patients [3] (Figure 2).

## Role of Exosomes in Cancer, Challenges, and Future Directions

Exosomes derived from cancer cells contain various molecules, including proteins, lipids, and nucleic acids, such as microRNAs, which can be transferred to recipient cells and alter their biological functions [4,43]. This can promote tumor growth, angiogenesis, invasion, and immune evasion [1,4]. However, studying the role of exosomes in cancer poses several challenges [1,4,13]. One of the significant challenges is the isolation and characterization of exosomes from complex biological fluids, such as blood, which contains a high number of non-exosomal particles [1,4]. Moreover, exosomes derived from different types of cancer cells may have different molecular compositions and functions [13]. Thus, developing specific strategies to target cancer-derived exosomes may be a promising therapeutic approach [13]. Another challenge is the lack of standardized protocols for the isolation and analysis of exosomes, making it difficult to compare results across different studies. Furthermore, the heterogeneity of exosomes and the lack of specific markers for their isolation and characterization also make it challenging to study their functions accurately [13,25]. Despite these challenges, the study of exosomes in cancer has great potential for identifying novel biomarkers and developing new therapeutic approaches. Future research directions include developing more specific and sensitive methods for the isolation and analysis of exosomes, identifying the molecular mechanisms underlying their functions, and exploring their potential as therapeutic targets for cancer treatment.

Interdisciplinary collaboration in exosome research fosters the development of promising cancer theragnostic for the future. Recent advancements include the integration of exosome-based cancer biomarker research with machine learning and artificial intelligence, paving the way for precision oncology. In the context of cancer, exosomes have been shown to play a critical role in various aspects of tumour progression, including tumour growth, invasion, and metastasis [13,44]. They can also contribute to drug resistance and immune evasion, making them an attractive target for therapeutic intervention [25].

## Concluding Remarks

Exosomes have emerged as a pivotal focus in the field of oncology, playing crucial roles in cancer metastasis. Exosomes are not directly used in artificial intelligence (AI) applications, but their analysis and interpretation can contribute to the development of AI tools for cancer diagnosis, prognosis, and treatment. Exosomes contain various biomolecules, including proteins, lipids, and nucleic acids, which can serve as potential biomarkers for cancer detection and monitoring. AI algorithms can be trained on large datasets of exosome-related molecular data to identify patterns and signatures that distinguish cancer from normal cells or predict disease progression and treatment response. For example, machine learning algorithms have been developed to analyze exosome-derived microRNA profiles and predict the stage and subtype of various cancer types, such as breast, lung, and prostate cancer. Other AI tools have been designed to analyze the lipid composition of exosomes and identify lipid markers associated with cancer progression and metastasis. Moreover, the transfer of exosomal cargo between cancer cells and their microenvironment can influence tumor growth and response to therapy. Understanding the mechanisms underlying exosome-mediated communication in cancer can help develop new therapeutic approaches, such as targeting specific exosomal proteins or miRNAs. AI algorithms can facilitate the identification of potential exosome-based targets and assist in the design of more effective therapies.

Challenges, such as standard isolation methods and toxicity, warrant further scientific investigation and clinical trials. Exosomes not only open up a vast realm of precision medicine but also serve as vital biomarkers for tracking cancer and testing new drugs to combat the disease. Owing to their remarkable stability and biocompatibility, exosomes hold promise as effective drug delivery systems for cancer treatment. The analysis of exosomes in cancer research can provide valuable data for AI applications in cancer diagnosis, prognosis, and treatment.

## Data Availability

No data was used for the research described in the article.
